# Mesoscale modelling of starch digestion

**DOI:** 10.1080/00268976.2024.2445770

**Published:** 2025-01-07

**Authors:** Muriel C. van der Laan, John R. Bows, Julia M. Yeomans

**Affiliations:** a Rudolf Peierls Centre for Theoretical Physics, University of Oxford, Oxford, UK; b PepsiCo R&D, Leicester, UK

**Keywords:** Mesoscale model, polymers, digestion, starch, phytochemicals

## Abstract

An idealised mesoscale model of the enzymatic digestion of starch modelled as a polymer aggregate is used to study the effect of various enzyme properties, such as the enzyme efficiency, range and radius, on the rate at which monomers are released from the aggregate. Depending on the enzyme efficiency the process is found to be either reaction- or diffusion-limited. Additionally the digestion rate is found to be proportional to the volume around each bond that is accessible to the enzyme, which is determined by the range and radius of the enzyme. Simulations of uniformly mixed susceptible and resistant polymers reveal no significant effect on the digestion of the susceptible polymers due to the presence of the resistant polymers.

## GRAPHICAL ABSTRACT

**Figure:**
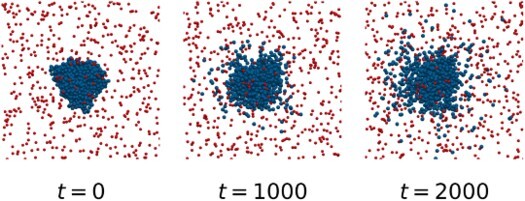

## 1. Introduction

It is clear that a balanced diet is essential for human health and well-being. Increasing insight into the behaviour of different food types during the digestive process is therefore of broad biomedical relevance. For example there is growing interest in the development of so-called functional foods, which are designed to be beneficial to health, by improving overall well-being or reducing the risk of certain diseases [1]. It is hoped that the development of computational models of digestive processes will lead to a better understanding of the behaviour of food as it is digested. Such models are however challenging to develop, as the digestion of food is characterised by the complex interplay of a range of chemical and physical processes taking place at multiple length scales [1,2].

Given the complex, multiscale nature of the process, computational models of the entire human digestive system are currently out of reach (and likely to remain so for the foreseeable future). In order to make progress, a range of models to study processes occurring at different length scales will be required [3]. Most existing computational models of digestive processes are based on continuum approaches. These include computational fluid dynamics simulations of the flow field in a simplified three-dimensional model of a human stomach [4,5] and a model of the transport and degradation of a bolus in the small intestine [6] based on a set of coupled differential equations. Such continuum models are well-suited for studying processes taking place at larger length scales, but any microscopic detail must be represented using approximate constitutive relations. In particular, this makes them unsuitable for studying the effect of different food structures on digestion. Fully representing all molecular detail would, on the other hand, quickly lead to infeasible simulations for any system of a realistic size.

Simulation methods which are well suited to studying processes occurring at intermediate length scales, at which structural information is important but including full molecular detail is too numerically intensive, are collectively termed mesoscale simulations. These approaches typically rely on simplified models of materials, coarse-graining over the molecular detail, but are still able to represent some structural features. Applications of mesoscale simulations to digestive process include studies of polymer aggregate digestion resulting from chemical bond breaking or shear flow [3] and the impact of villi motion on the transport of nutrients to the intestinal wall [7,8].

Here we focus on using a mesoscale model to study the enzymatic digestion of starch, one of the main components of the human diet. It consists of two main components: amylose and amylopectin, both of which are polymers of *α*-glucose [9,10]. Starch is broken down into maltose, a disaccharide consisting of two *α*-glucose units, by the enzyme *α*-amylase in the mouth and in the small intestine [11]. As the release of glucose from starchy foods is associated with obesity, diabetes and other metabolic disorders, there is a strong interest in manipulating the rate of enzymatic hydrolysis in starch in order to control the rise in blood glucose concentrations and the associated insulin response after the ingestion of a meal [11].

We model the digestion of starch using a simplified polymer model in which the polymers are represented by chains of beads connected by harmonic potentials. Extending an earlier model of polymer aggregate digestion [3] we explicitly add enzyme molecules which break nearby polymer bonds to the simulation. This allows us to study the effect of various enzyme parameters, such as the efficiency, size and interaction range of the enzyme on the rate of digestion of the polymer aggregate.

Inevitably any tractable model of the complex digestion process is highly idealised. Our paper represents a first step towards building mesoscale models that can be employed, along with other numerical approaches and experiments, to provide tools to understand digestion.

## 2. Methods

Our simulations consist of a coarse-grained polymer model representing the starch, simplified enzyme molecules and hydrodynamic interactions modelled using multi-particle collision dynamics. We will first describe each of the components of the model and how they interact with each other, and then summarise the initial conditions and system parameters.

The starch is modelled as a network of interacting bead-spring polymers. The radius of the beads is given by $r_{\mathrm{poly}}$. Neighbouring beads within a polymer interact through a harmonic potential given by

(1) $$u_{\mathrm{bond}}=\frac{1}{2}k_{\mathrm{bond}}(r-l_{0}{)}^{2},$$

where *r* is the distance between the beads, $l_{0}$ is the equilibrium bond length, which is taken to be twice the bead radius $r_{\mathrm{poly}}$, and $k_{\mathrm{bond}}=10^{5}k_{B}T$, where $k_{B}$ is the Boltzmann constant and *T* is the temperature. Any beads not connected by a harmonic bond interact through a potential

(2) $$u=u_{\mathrm{LJ}}+u_{\mathrm{offset}}.$$

$u_{\mathrm{LJ}}$ is a truncated Lennard-Jones potential, given by

(3) $$u_{\mathrm{LJ}}=\left\{ \begin{array}{ll} \epsilon +4\epsilon \left({\left(\frac{\sigma^{*}}{r}\right)}^{12}-{\left(\frac{\sigma^{*}}{r}\right)}^{6}\right) & \mathrm{if}\;r\leq r_{\mathrm{cut}-\mathrm{off}}\\ 0 & \mathrm{if}\;r>r_{\mathrm{cut}-\mathrm{off}} \end{array}\right.,$$

where *ϵ* is the interaction strength. $u_{\mathrm{offset}}$ is a small correction added to ensure that the force (i.e. the derivative of the potential) is the same on both sides of the cut-off, as discontinuities in the force can lead to numerical instabilities [12]. This correction is given by

(4) $$u_{\mathrm{offset}}=\left\{ \begin{array}{ll} -\frac{r^{2}}{2r_{\mathrm{cut}-\mathrm{off}}}{\frac{\partial u_{\mathrm{LJ}}}{\partial r}|}_{r=r_{\mathrm{cut}-\mathrm{off}}} & \mathrm{if}\;r\leq r_{\mathrm{cut}-\mathrm{off}}\\ 0 & \mathrm{if}\;r>r_{\mathrm{cut}-\mathrm{off}} \end{array}\right..$$

For the polymer–polymer interactions we set $\sigma^{*}=\sigma$, where *σ* is the minimum distance between two polymer beads, i.e. $2r_{\mathrm{poly}}$, and $r_{\mathrm{cut}-\mathrm{off}}=2.5\sigma$, which gives a potential that is repulsive at short distances, but attractive at intermediate distances. In the absence of attraction, the aggregate would disperse due to diffusion without any enzyme activity: The interaction strength is tuned so that aggregates of long polymers remain stable against diffusion, but individual monomers disperse.

The enzyme molecules are modelled as spherical beads of radius $r_{\mathrm{enzyme}}$, which interact with other enzyme molecules and with polymer beads through the truncated Lennard-Jones potential given in equation (3). For the enzyme–enzyme and enzyme–polymer interactions we set $\sigma^{*}=\sigma /2^{\frac{1}{6}}$ and $r_{\mathrm{cut}-\mathrm{off}}=\sigma$, where *σ* is the minimum distance between the beads i.e. $2r_{\mathrm{enzyme}}$ for enzyme–enzyme interactions and $r_{\mathrm{enzyme}}+r_{\mathrm{poly}}$ for enzyme–polymer interactions. This leads to a fully repulsive interaction.

At each time step, any polymer bonds that are within a distance $R_{\mathrm{enzyme}}$ of an enzyme molecule are broken with probability $p_{\mathrm{break}}$. When the bond between two monomers is broken, there is no longer a harmonic potential connecting them and the monomers instead interact through the off-set Lennard-Jones potential given in (2). The breakage probability $p_{\mathrm{break}}$ represents the efficiency of the enzyme.

The polymers and enzymes are embedded in an effective fluid modelled using multi-particle collision dynamics (MPCD). In the MPCD algorithm the fluid consists of point particles which undergo alternating free streaming and collision steps [13]. In the collision step the fluid particles are divided into cells within which momentum is exchanged according to a collision rule which ensures that the total linear and angular momentum in each collision cell is conserved. To couple the polymers and enzymes to the fluid we include them in the collision step, by sorting them into cells along with the fluid particles and including them in the collision rule [13,14]. During streaming steps, the trajectories of the polymer and enzyme beads are calculated using the velocity Verlet algorithm.

We start the simulation with all polymers located in a sphere of radius $r_{\mathrm{agg}}=10r_{\mathrm{poly}}$ and all enzymes outside the sphere. The monomer positions for each polymer are determined by generating random walks with step size $l_{0}$ until a configuration is found in which all monomers are contained within the sphere. We run the system for an equilibration period during which overlaps between monomers are removed and thermodynamic equilibrium is achieved. During this time, the system evolves as described above, but the enzymes do not break any polymer bonds.

We perform the simulations in a box consisting of 40×40×40 cubic MPCD collision cells with a side length equal to the monomer diameter $2r_{\mathrm{poly}}$. Periodic boundary conditions are applied at the edges of the box. Each collision cell contains on average 10 fluid particles, which have a mass equal to one tenth of the monomer and enzyme masses. We perform collision and streaming steps at time intervals of 0.02, and perform 50 velocity Verlet integration steps updating the positions of the polymer beads and enzymes during each streaming step. At these parameter values both disconnected monomers and the enzyme molecules have a diffusion coefficient of $(4.48\pm 0.16)\cdot 10^{-2}$ in simulation units (in which $r_{\mathrm{poly}}=1$). Other simulation parameters are listed in Table 1.

**Table 1.** Simulation parameters.

Parameter	Significance	Default value(s)
$N_{\mathrm{poly}}$	Number of polymers in the aggregate	40
$L_{\mathrm{poly}}$	Number of beads in each polymer	20
$N_{\mathrm{enzyme}}$	Number of enzyme molecules	500
$R_{\mathrm{enzyme}}$	Enzyme range: distance within which enzymes are able to break polymer bonds	$4r_{\mathrm{poly}}$
$r_{\mathrm{enzyme}}$	Enzyme radius	$r_{\mathrm{poly}}$
$p_{\mathrm{break}}$	Enzyme efficiency: probability a polymer bond gets broken if it is within range of an enzyme molecule	1.0 or 0.001
$r_{\mathrm{agg}}$	Radius of the polymer aggregate	$10r_{\mathrm{poly}}$

Note: The default value is used unless otherwise stated. Distances are given relative to $r_{\mathrm{poly}}$, the polymer bead radius.

## 3. Results

We first describe how we determine appropriate values for the length of the equilibration period and the strength of the Lennard-Jones interaction between monomers. Then we discuss how to analyse the simulation data to allow comparison to experimental results. We then describe the effect of various enzyme properties on the digestion rate. Finally we discuss the digestion of aggregates consisting of a mix of polymers that are either resistant or susceptible to the enzyme.

**Figure 1.**
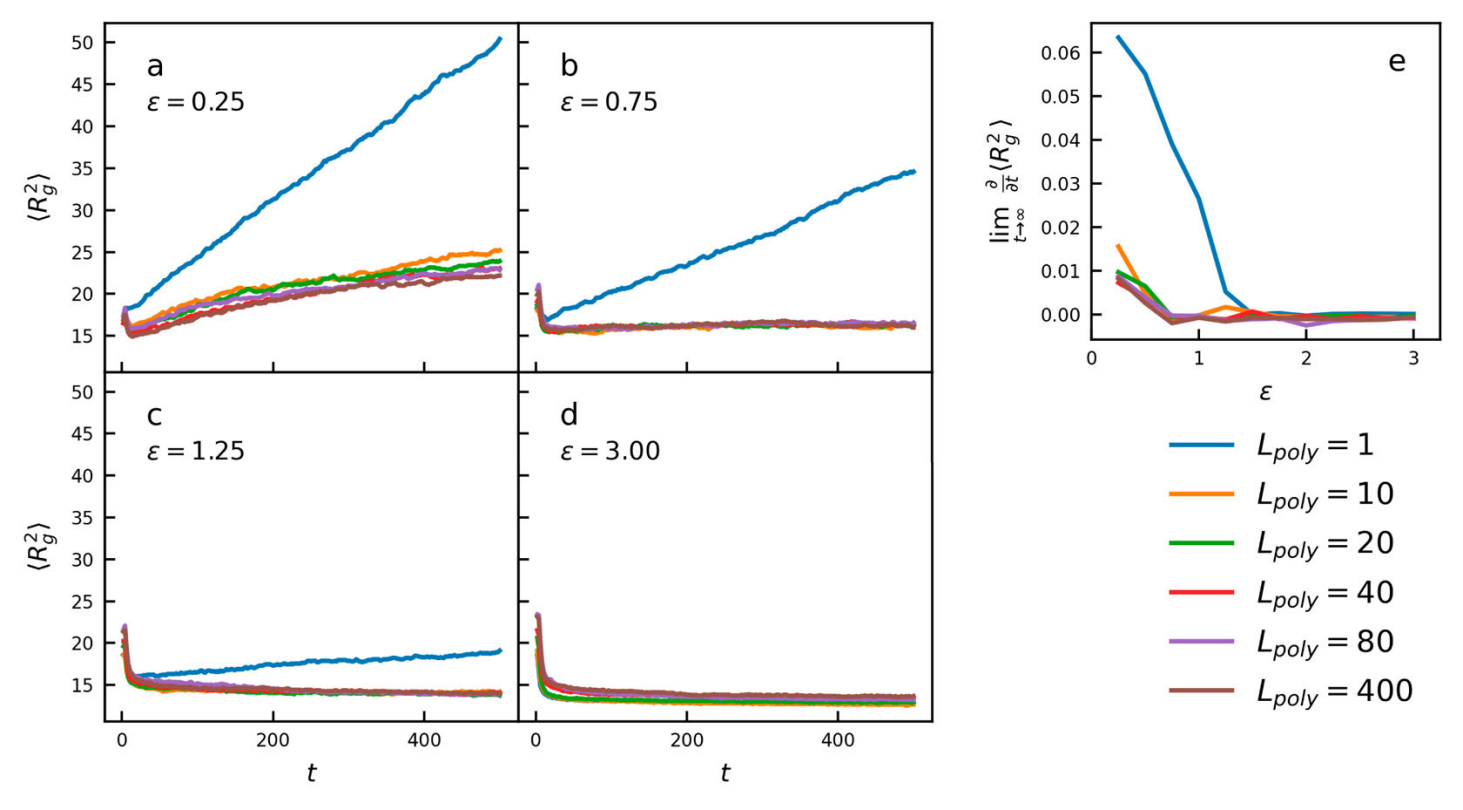
(a–d) Ensemble-averaged squared gyration radius $\left\langle R_{g}^{2}\right\rangle$ as a function of time *t* for different polymer lengths $L_{\mathrm{poly}}$ and interaction strengths *ϵ*. The polymer length was varied by keeping the total number of monomers constant at 400 and varying the number of polymers in the aggregate. (e) Long-time expansion rate as a function of the interaction strength for different polymer lengths. These plots were obtained by fitting a straight line to the last third of the gyration radius traces.

To determine an appropriate length for the equilibration period and a suitable value for the interaction strength between monomers, we calculate the aggregate gyration radius, given by

(5) $$R_{g}^{2}(t)=\frac{1}{N}\sum_{i}^{N}(r_{i}-r_{\mathrm{CM}}{)}^{2}$$

where *N* is the total number of monomers, $r_{i}$ is the position of the *i*th monomer and $r_{\mathrm{CM}}$ is the position of the centre of mass of the aggregate. Figure 1(a–d) show the time evolution of the ensemble-averaged gyration radius for aggregates of polymers of different lengths and at different interaction strengths. No enzymes were present in these simulations.

At first the aggregates expand rapidly as the short-range repulsive interaction between the monomers removes any overlaps in the initial monomer positions. This initial expansion is followed by a period of contraction as the long-range attractive interactions take over and the aggregate relaxes to an equilibrium size. Depending on the length of the polymers and the strength of the monomer-monomer interaction, the gyration radius then either stays at a constant value, indicating the aggregate is stable against diffusion, or slowly increases, indicating that the polymers are dispersing. We set the length of the equilibration period to 100 (5000 time steps) to ensure that the initial rapid expansion and contraction are concluded within the equilibration period and that the aggregate, if it is stable, has reached an equilibrium. Figure 1(e) shows the long-time expansion rate $\lim_{t\to \infty }\frac{\partial }{\partial t}\langle R_{g}^{2}\rangle$ as a function of the polymer length $L_{\mathrm{poly}}$ and the interaction strength *ϵ*. In this figure non-zero values indicate an aggregate that is dispersing, while zero values indicate a stable aggregate. Individual monomers ( $L_{\mathrm{poly}}=1$) disperse for ϵ≲1.0 whereas polymer chains disperse for ϵ≲0.5. In the light of these results we set the interaction strength *ϵ* to 0.75 in the rest of our simulations, so that the aggregate is stable against diffusion, but monomers that have been disconnected will disperse.

Experimental data on starch digestion is typically analysed by assuming first order kinetics, in which the reaction rate is proportional to the amount of undigested starch present. The amount of maltose, which corresponds to the monomers in our simulations, released over time is then given by

(6) $$N(t)=N_{\infty }(1-e^{-\mathrm{kt}})$$

where $N_{\infty }$ is the total amount of maltose released at long times and *k* is a rate constant. In order to compare our results with experimental data we track the number of monomers released over time. A monomer is counted as released when both the harmonic bonds connecting it to its neighbours along a polymer chain are broken. We note, however, that visual inspection of the simulation results suggests that the majority of bonds are broken near the surface of the aggregate, which would imply the rate of digestion is proportional to the surface area of the aggregate, rather than its volume, giving equation (A4) (derived in Appendix A) for the number of monomers released as a function of time, instead of equation (6). Equations (6) and (A4) can be made to give equally good fits by adjusting the parameters *k* and $k^{\prime }$, which control the overall timescale of digestion and in many cases where there is no a priori estimates of the digestion rate it is impossible to distinguish between the two. We therefore choose to fit our results to the first order kinetics result given in equation (6) in accordance with the usual approach in the literature.

**Figure 2.**
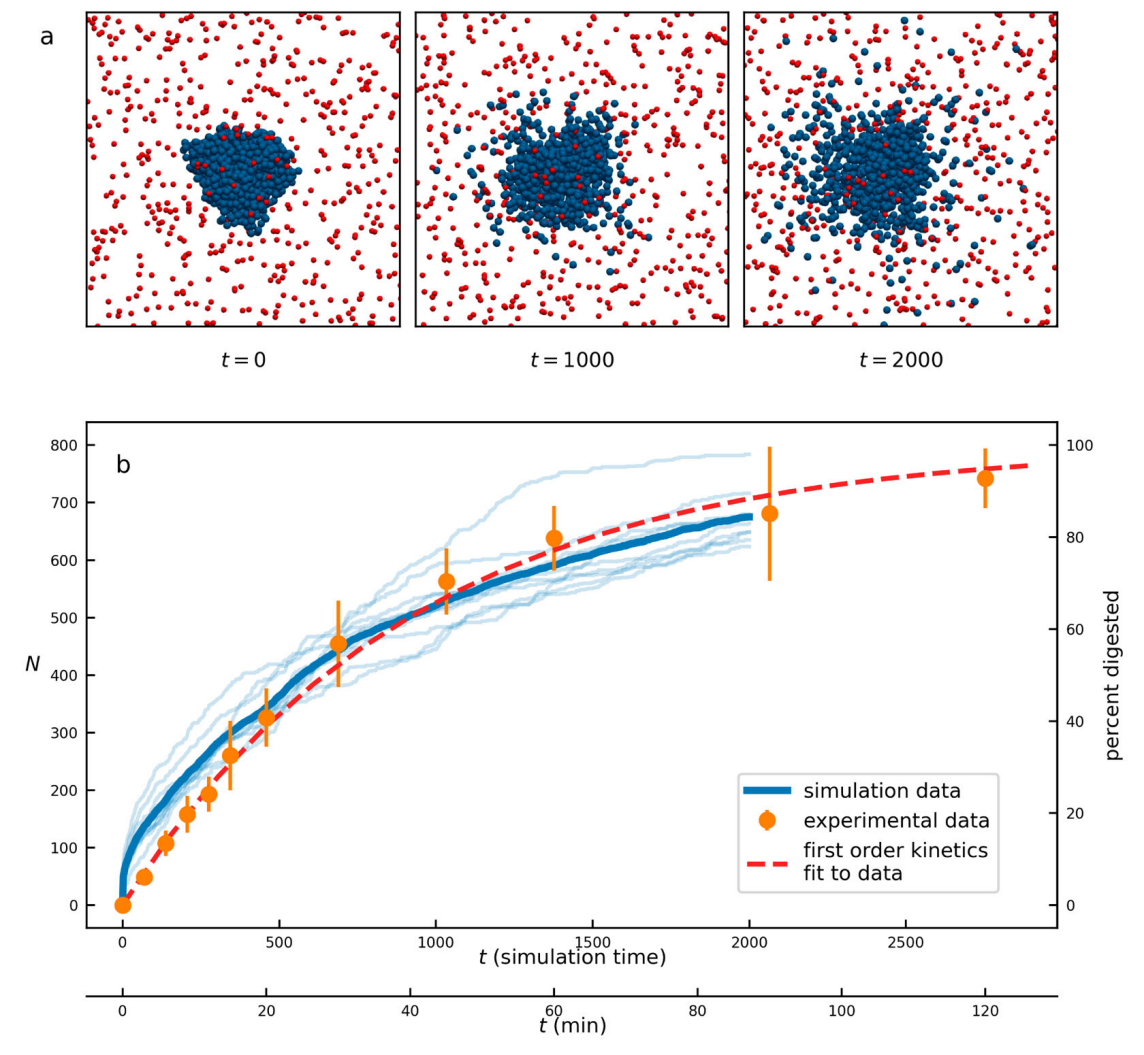
(a) Snapshots of the evolution of a polymer aggregate under the influence of enzymatic action. The parameters used in the simulation are those given in Table 1 with $p_{\mathrm{break}}=1.0$. Polymers are shown in blue, enzymes are shown in red. Enzymes are shown reduced in size for visual clarity. (b) Simulations showing the number of monomers released from a polymer aggregate under the influence of enzymatic action *N* as a function of time *t* compared to experimental data [15]. Ten individual simulations (light blue lines) and their mean (darker blue) are shown. The simulation time is given as the number of time steps since the start of the simulation. For the experimental data, the time is given in minutes. The different timescales are scaled according to equation (7) in order to make the first order kinetics fits for the simulation data and experimental data coincide.

Snapshots showing the evolution of the aggregate with time for the default parameters given in Table 1 are shown in Figure 2(a). Figure 2(b) shows the number of monomers released as a function of time for the same parameter values. Equation (6) provides a good fit for the simulations. Also shown is sample experimental data [15] for maltose concentration as a function of time. We can use the first order kinetics digestion rates obtained by fitting equation (6) to both the simulation results and the experimental data to relate the number of time steps in the simulation to the experimental time:

(7) $$t_{\exp}=\frac{k_{\mathrm{sim}}}{k_{\exp}}t_{\mathrm{sim}}.$$

Next we studied how the digestion rate and total number of monomers released depend on the enzyme efficiency $p_{\mathrm{break}}$, the number of enzyme molecules $N_{\mathrm{enzyme}}$, the enzyme range $R_{\mathrm{enzyme}}$ and the enzyme radius $r_{\mathrm{enzyme}}$. For each of these parameters we ran five simulations with different random seeds for several different values of that parameter while all other parameters were kept constant at the default values listed in Table 1. We then calculated the digestion rate (*k*) and total number of monomers released over time ( $N_{\infty }$) for each set of parameters by fitting equation (6) to data from each of the five simulations and taking the mean of the fitting parameters obtained.

**Figure 3.**
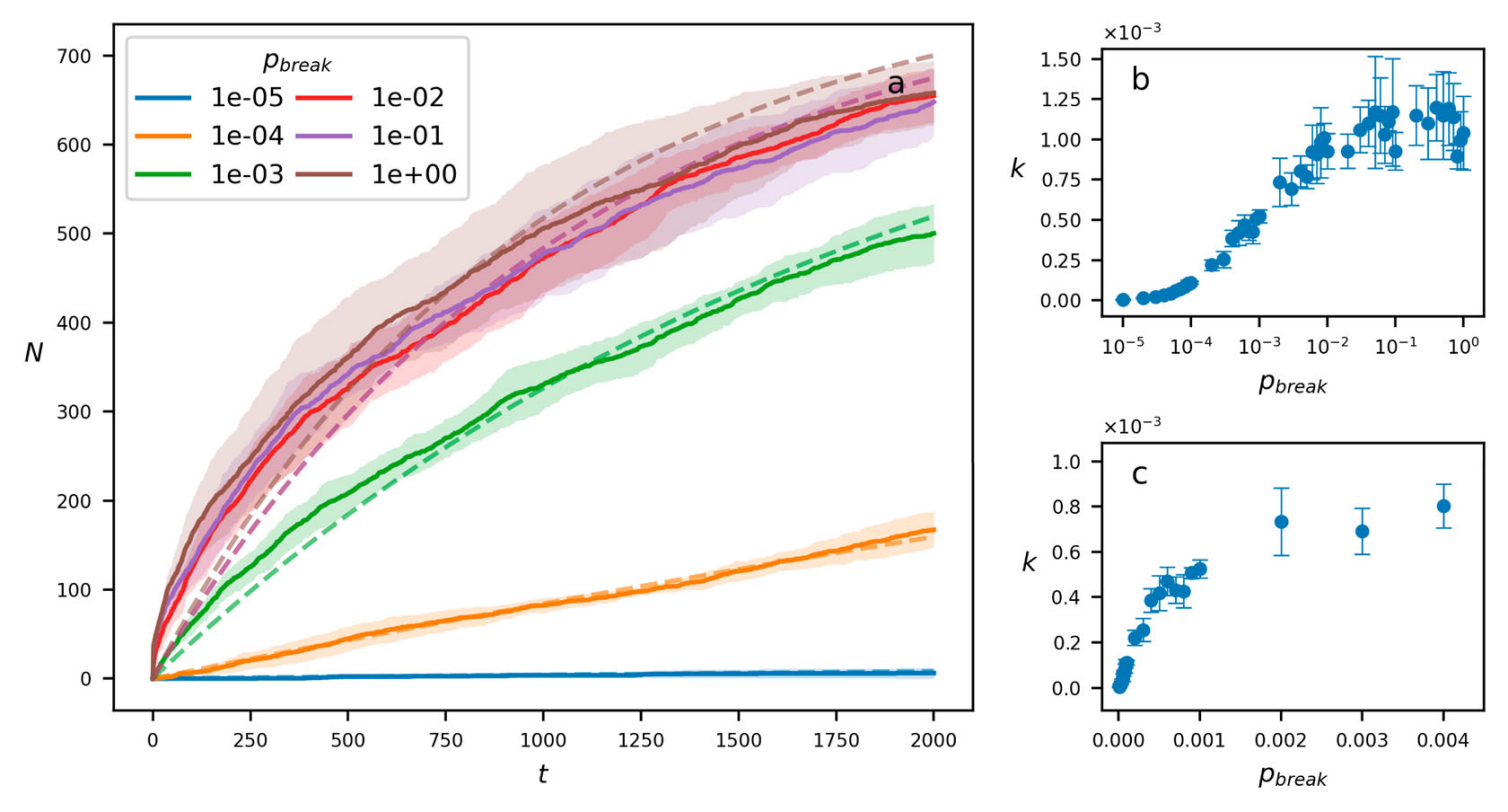
(a) Number of monomers released *N* over time *t* at different enzyme efficiencies $p_{\mathrm{break}}$. The solid lines show the ensemble average over 5 simulations, with the shaded area showing the standard deviation. The dashed lines are fits to equation (6). These fits fix $N_{\infty }$ at the total number of monomers present in the simulation as at low enzyme efficiencies the timespan of our simulations is not long enough to accurately determine $N_{\infty }$. (b) Digestion rate *k* as a function of enzyme efficiency $p_{\mathrm{break}}$. $p_{\mathrm{break}}$ is shown on a log scale. (c) Digestion rate *k* as a function of enzyme efficiency $p_{\mathrm{break}}$ for low $p_{\mathrm{break}}$, shown on a linear scale.

Figure 3 shows the number of monomers released over time for simulations performed with varying enzyme efficiencies $p_{\mathrm{break}}$ together with best fits to equation (6). These fits assume that $N_{\infty }$ is the total number of monomers present in the simulation, as at low enzyme efficiencies the length of the simulations is not sufficient to determine $N_{\infty }$. Figure 3(b,c) show the digestion rate at different values of $p_{\mathrm{break}}$. At low $p_{\mathrm{break}}$, this increases approximately linearly with $p_{\mathrm{break}}$ whereas at higher $p_{\mathrm{break}}$ it plateaus to a constant value. This corresponds to a transition from a reaction-limited process, where the enzyme is not able to break all bonds that are within its range before it diffuses away, to a diffusion-limited process, where the enzyme quickly breaks all bonds within its range and then has to wait until new bonds come into range.

**Figure 4.**
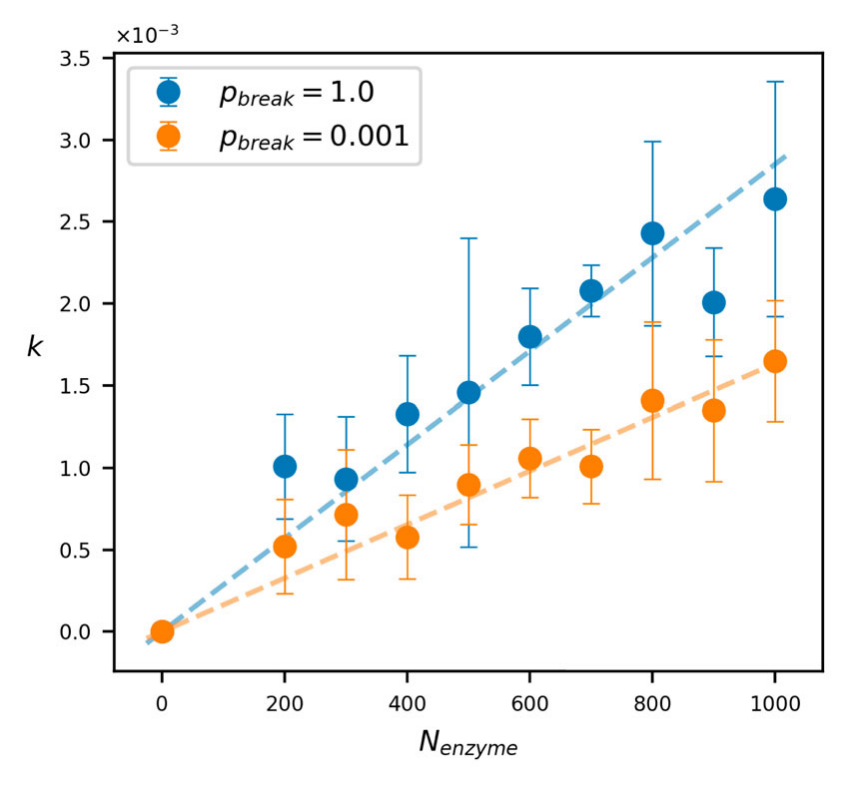
Digestion rate *k* as a function of the number of enzymes present in the simulation.

Figure 4 shows the digestion rate for varying numbers of enzyme molecules. These simulations were run with both a low enzyme efficiency ( $p_{\mathrm{break}}=0.001$) and a high enzyme efficiency ( $p_{\mathrm{break}}=1.0$). As expected the digestion rate is proportional to the number of enzyme molecules present in both cases.

**Figure 5.**
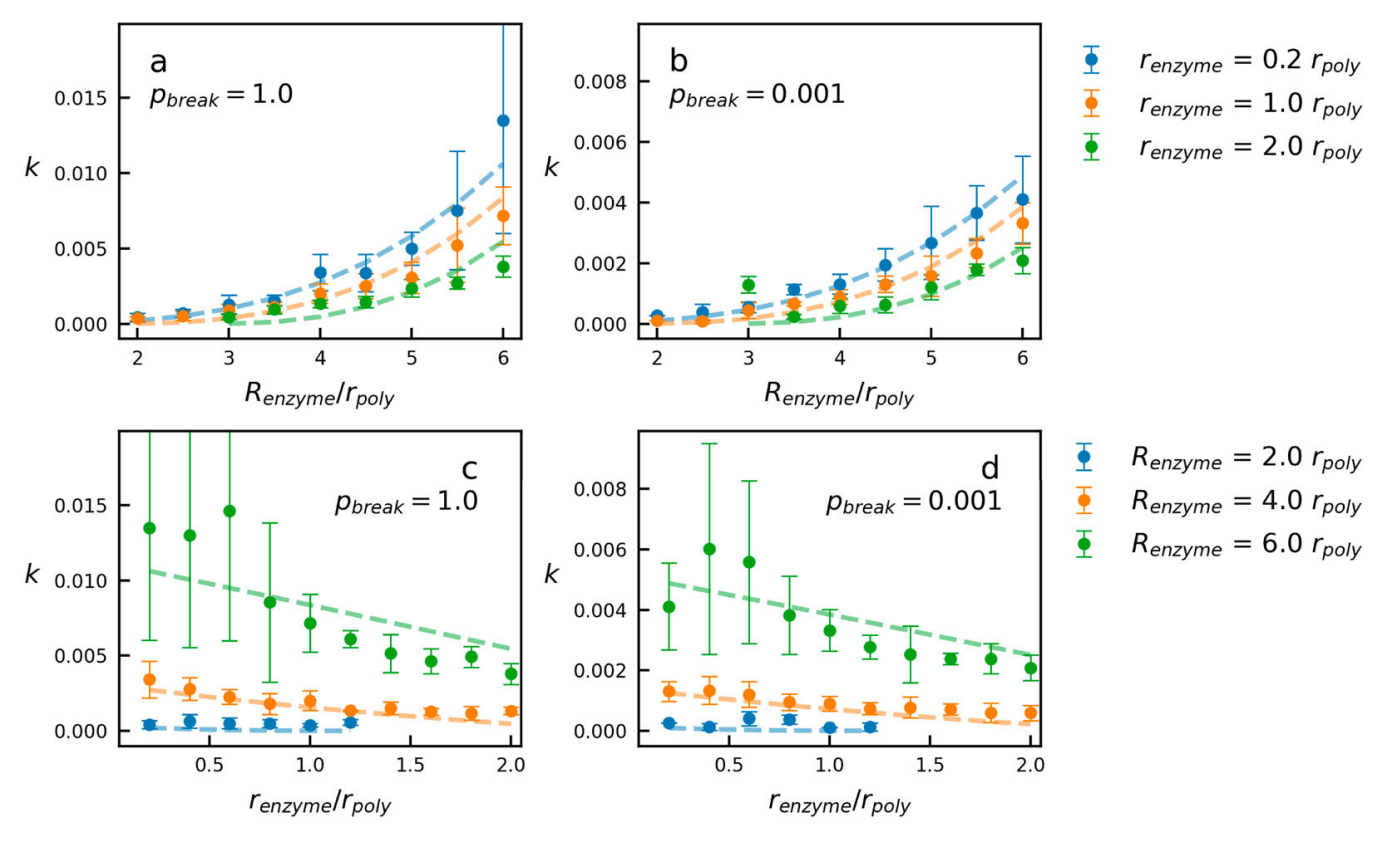
(a,b) Digestion rate *k* as a function of the enzyme range $R_{\mathrm{enzyme}}$ at different enzyme radii for diffusion-limited ( $p_{\mathrm{break}}=1.0$) and reaction-limited ( $p_{\mathrm{break}}=0.001$) enzymes. (c,d) Digestion rate *k* as a function of the enzyme radius $r_{\mathrm{enzyme}}$ at different enzyme ranges for diffusion-limited ( $p_{\mathrm{break}}=1.0$) and reaction-limited ( $p_{\mathrm{break}}=0.001$) enzymes. The enzyme radius is given in terms of the monomer radius $r_{\mathrm{poly}}$. (a–d) The dashed lines show the best fit assuming a linear relationship between the available reaction volume and the digestion rate and using the fitting parameters from Figure 6.

**Figure 6.**
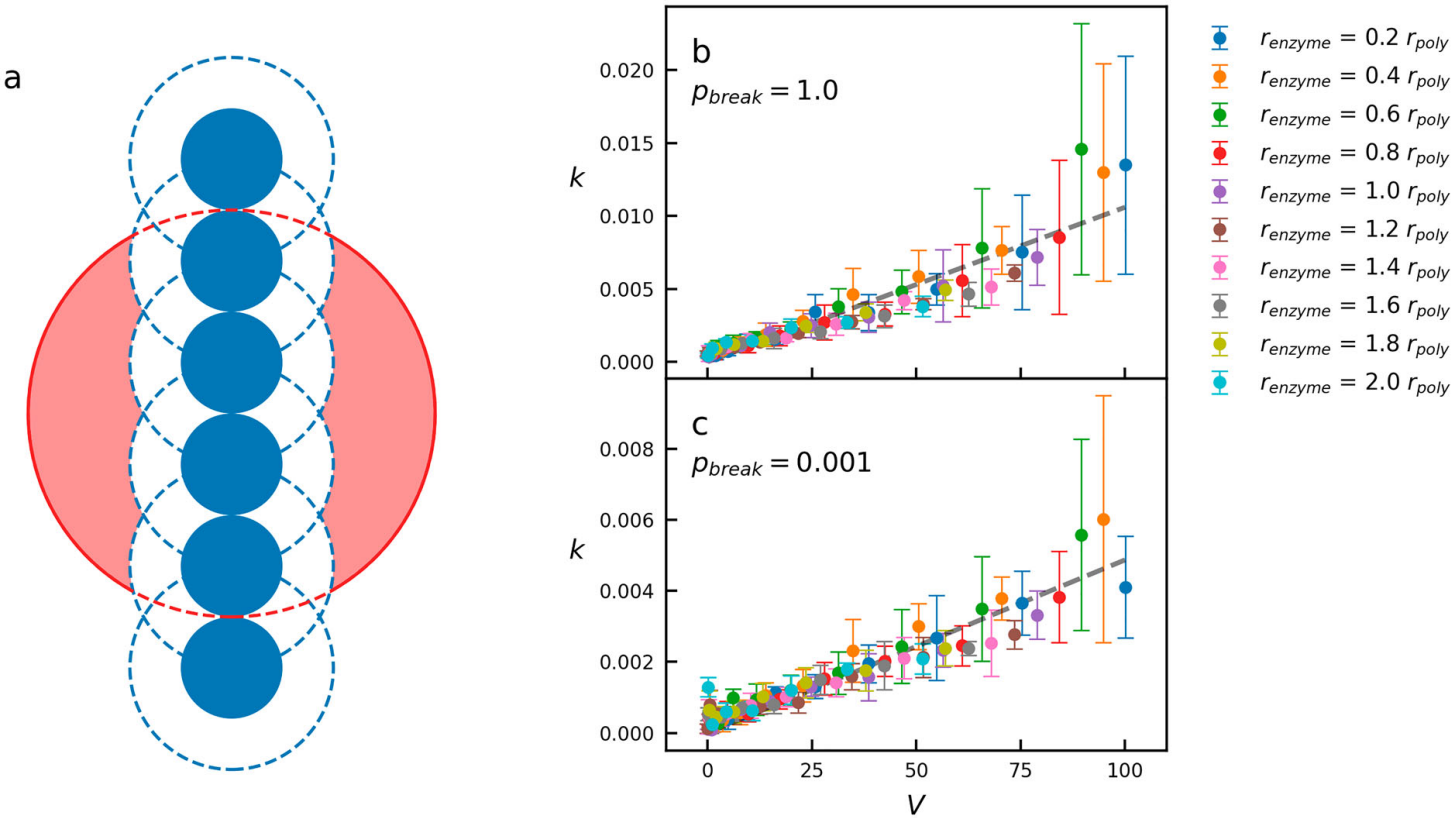
(a) Available reaction volume around a bond in the centre of a polymer. The bond is surrounded by a sphere of radius $R_{\mathrm{enzyme}}$, the enzyme range, (shown in red) in which the enzyme would be close enough to break the bond. Each monomer blocks out a sphere of radius $r_{\mathrm{poly}}+r_{\mathrm{enzyme}}$ around itself (shown in blue). This leaves the red shaded volume, *V*, available for reactions to take place. (b,c) Digestion rate *k* as a function of the available reaction volume *V* for diffusion-limited ( $p_{\mathrm{break}}=1.0$) and reaction-limited ( $p_{\mathrm{break}}=0.001$) enzymes. The different colours correspond to different enzyme radii (see legend). The available volume is calculated for polymers of length 20, using Monte Carlo integration, averaged over each bond in the polymer and 500 different polymer conformations. The dashed line shows the best fit assuming a linear relationship between *k* and *V*.

Figure 5 shows the digestion rate as a function of the enzyme range $R_{\mathrm{enzyme}}$ and the enzyme radius $r_{\mathrm{enzyme}}$. The digestion rate increases for larger enzyme ranges and smaller enzyme radii. These results are best understood in terms of the available volume for reactions. Each bond is surrounded by a sphere of radius $R_{\mathrm{enzyme}}$ in which enzymes are close enough to break this bond. The larger the sphere, the higher the probability that the bond will be broken in a given time step. However, not all of this volume is accessible to the enzyme, as some of it will be blocked by the monomers connected by the bond and their neighbours. The smaller the enzyme, the closer it will be able to get to the monomers, and the more likely it is to break bonds. A diagram showing the volume around a given bond that is both accessible to enzymes and within range for enzymes to break the bond is shown in Figure 6(a). Figure 6(b,c) show the relationship between this volume and the digestion rate for different enzyme ranges and enzyme radii for both diffusion- and reaction-limited enzymes. In both cases, the relationship is indeed linear.

**Figure 7.**
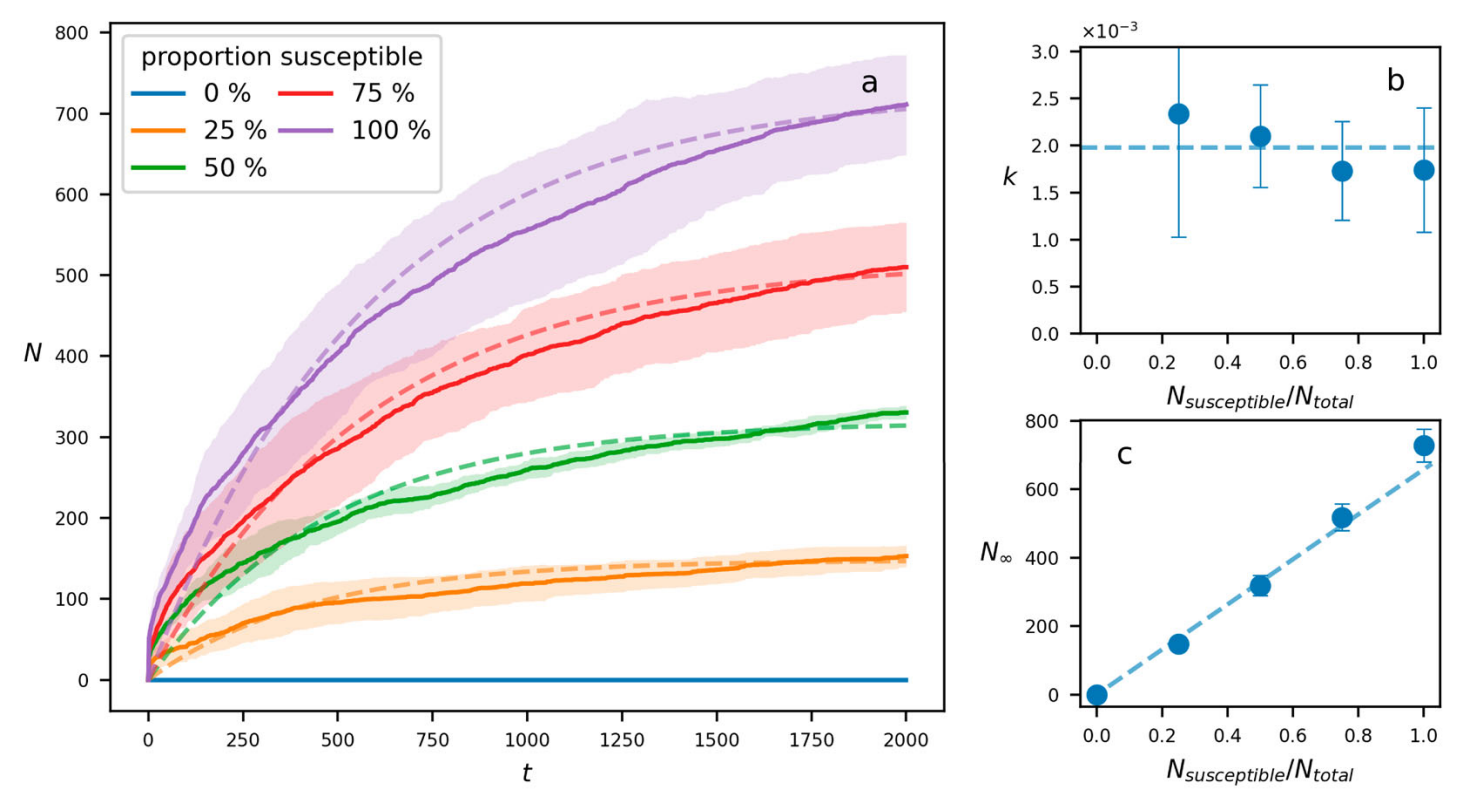
(a) Number of monomers released *N* over time *t* for aggregates containing different proportions of enzyme-resistant polymers. The total number of polymers in the aggregate is kept constant. The solid lines show the ensemble average over 5 simulations, with the shaded area showing the standard deviation. The dashed lines are a fit to equation (6). (b) Digestion rate *k* as a function of the fraction of susceptible polymers in the aggregate. The dashed line shows the average value of *k* over all values of $N_{\mathrm{susceptible}}/N_{\mathrm{total}}$. (c) The number of monomers released at long times $N_{\infty }$ as a function of the fraction of susceptible polymers in the aggregate. The dashed line is a linear fit to the data.

Simulations were also run with a mixture of susceptible polymers, with bonds that are broken when they are near an enzyme molecule, and resistant polymers, whose bonds never get broken. In these simulations the susceptible and the resistant polymers are distributed evenly within the aggregate. Figure 7(a) shows the number of monomers released over time for different ratios of resistant and susceptible polymers, together with the best fits to the rate equation (6). Figure 7(b,c) show the dependence of the digestion rate and the total number of monomers released on the proportion of susceptible polymers in the aggregate. The total number of monomers released increases linearly as the proportion of susceptible polymer increases as expected. Additionally, the digestion rate does not depend on the proportion of susceptible polymers indicating that this is not affected by the presence of the resistant polymers.

## 4. Discussion

We constructed a mesoscale model of the digestion of starch by *α*-amylase, consisting of a course-grained polymer aggregate and simplified enzyme molecules. Including explicitly modelled enzymes in the model allowed us to study the effect of various enzyme properties, such as the enzyme efficiency, enzyme range and enzyme radius, on the rate at which monomers are released from the aggregate. At low enzyme efficiencies the process was found to be reaction-limited, with the digestion rate increasing with increasing enzyme efficiency, whereas at high enzyme efficiencies the digestion rate plateaued, indicating that the digestion had become diffusion-limited. Additionally the digestion rate was found to be proportional to the volume around each bond that is available to the enzyme. This volume is determined by the enzyme range and radius.

We also studied the digestion of aggregates consisting of a mixture of polymers that are susceptible to enzyme action and polymers that are resistant to enzyme action, finding that the total number of monomers released is proportional to the proportion of susceptible polymers in the aggregate, but the digestion rate is independent of the proportion of susceptible polymers. This indicates that the presence of resistant polymers did not affect the digestion of the susceptible polymers.

Due to computational limitations we have had to simplify the problem of starch digestion in a number of ways. First of all, in order to have the simulations finish in a reasonable amount of time we have had to increase the number of enzyme molecules, which gives an enzyme-to-starch ratio that is considerably higher than it would be in realistic circumstances. We studied the impact of reducing the number of enzymes (see Figure 4) and found that the rate of digestion is simply proportional to the number of enzyme molecules, which indicates that individual enzymes are equally effective at different enzyme concentrations. We expect this trend will continue to even smaller numbers of enzymes. Secondly, we have neglected many of the details of the chemical reaction by which *α*-amylase breaks the bonds between glucose units, as this reaction takes place at length and time scales below the resolution of our simulation. Finally, starch granules have a complex hierarchical structure, consisting of alternating amorphous growth rings, in which the polymers are arranged in a disordered fashion, and semi-crystalline growth rings, which consist of alternating disordered and crystalline layers [10]. We are limited in the size of the aggregate that can be feasibly simulated, and so are not able to represent this structure in its entirety. However, in future work we may focus on simulating the digestion of individual parts of this structure.

So far we have mainly considered the effect of different enzyme properties on the digestion rate. In future work we will focus on the effect of different polymer properties [11]. Experimental evidence suggest that the amorphous regions in starch are digested faster than the semi-crystalline regions [16] and it is thought that this is largely due to the increased density in the ordered regions. In future work it would be interesting to vary polymer density and alignment independently to help determine which feature has most effect on digestion. Experiments have also shown that highly-branched starch is digested more slowly than less branched starch [17] and it will also be interesting to use the mesoscale approach to study the effects of polymer architecture on the digestion rate. Other directions are to model variations in the digestion rate due to physical barriers such as cell walls, protein networks and dietary fibre protecting the starch, or to consider the release of nutrients such as phytochemicals from the starch matrix.

Mesoscale models can predict trends and help to interpret the processes contributing to starch digestion. More quantitative approaches will require multiscale models, incorporating data from microscopic models that incorporate the structure of starch in more detail into the mesoscopic simulations.

## Appendix. Analytical results

In the literature, starch digestion is typically analysed using first order kinetics, which assumes that the rate of digestion is proportional to the amount of undigested starch present. This leads to equation (6) given in the main text. However, visual inspection of our simulation results shows that the enzyme molecules do not penetrate into the aggregate to a significant degree, and so polymers in the centre of the aggregate are not immediately available to be digested. By assuming that the rate of digestion must be instead be proportional to the surface area of the aggregate, we can again derive an expression for the amount of monomers released as a function of time. The rate of change of the volume of the aggregate V(r), where *r* is the radius of the aggregate, is proportional to its surface area A(r):

(A1)
$$\frac{dV(r)}{dt}\propto -A(r),$$

which gives

(A2)
$$\frac{dr}{dt}=-C$$

for some constant of proportionality *C*. This can be solved to obtain the following:

(A3)
$$r(t)=\left\{ \begin{array}{ll} r(0)-\mathrm{Ct} & \mathrm{for}\;\mathrm{Ct}\leq r(0)\\ 0 & \mathrm{for}\;\mathrm{Ct}>r(0) \end{array}\right.,$$

so that the number of monomers released as a function of time (proportional to V(0)−V(t)) is given by:

(A4)
$$N(t)=\left\{ \begin{array}{ll} N_{0}(1-(1-k^{\prime }t{)}^{3}) & \mathrm{for}\;k^{\prime }t\leq 1\\ N_{0} & \mathrm{for}\;k^{\prime }t>1 \end{array}\right..$$
